# Die Schmerzasymbolie – um 1930 von Paul F. Schilder entdeckt und heute fast vergessen?

**DOI:** 10.1007/s00482-020-00447-z

**Published:** 2020-02-25

**Authors:** Martin Jahn, Holger Steinberg

**Affiliations:** 1 Forschungsstelle für die Geschichte der Psychiatrie, Klinik und Poliklinik für Psychiatrie und Psychotherapie, Medizinische Fakultät, Universität Leipzig, Semmelweisstraße 10, 04103 Leipzig, Deutschland; 2 Neurologisches Rehabilitationszentrum Leipzig, Bennewitz, Deutschland

**Keywords:** Geschichte der Neurologie, 20. Jahrhundert, Schmerzwahrnehmung, Agnosie, Erstbeschreibung, History of neurology, Twentieth century, Pain asymbolia, Agnosia, First description

## Abstract

Paul Ferdinand Schilder (1886–1940) blieb der Nachwelt insbesondere in Erinnerung als Psychoanalytiker und Psychotherapeut. Allerdings forschte er auch auf neurowissenschaftlichem Gebiet umfassend und innovativ, so gilt er z. B. als Erstbeschreiber der nach ihm benannten Schilder-Krankheit. Im Mittelpunkt der hier vorliegenden Studie steht das ebenfalls von ihm erstbeschriebene Störungsbild der Schmerzasymbolie, das heute eher wenig bekannt ist und als selten gilt. Dabei handelt es sich um eine zentral bedingte Beeinträchtigung des Schmerzerlebens, die charakterisiert ist durch das Fehlen negativ-emotionaler Wahrnehmungen. Basis für Schilders Entdeckung und differenzialdiagnostische Abgrenzung der Schmerzasymbolie war die ausführliche Untersuchung von elf Krankengeschichten zwischen 1928 und 1930. Seine diesbezüglichen Publikationen kennzeichnen Akribie, vorwärtsgewandtes Denken und kritische Selbstreflexion. Er ordnete die Schmerzasymbolie nosologisch den Agnosien zu und integrierte sie in das Konzept des Körperschemas, das zeitlebens ein zentrales Thema seines wissenschaftlichen Wirkens war. Dieser Artikel geht auch auf die Frage ein, inwieweit Schilders Annahmen noch heute gültig sind und welche Konsequenzen sich hieraus ergeben könnten.

## Abstract (en)

Paul Ferdinand Schilder was born in Vienna in 1886 and died in New York in 1940. Today he is remembered particularly as a psychoanalyst and a psychotherapist. His research in neuroscience, however, was also both comprehensive and innovative. For example, he is considered to be the first to describe Schilder’s disease, which was named after him. This article focuses on pain asymbolia, which was also first described by Schilder, and is currently little known and considered to be rarely encountered. Pain asymbolia is a central impairment of pain experience with no negative affective-emotional component. The basis of Schilder’s discovery and the differential diagnosis of pain asymbolia was the detailed examination of eleven medical cases between 1928 and 1930. His publications on the condition are characterized by meticulousness, progressive thinking and critical reflection. He nosologically assigned pain asymbolia to the group of agnosias and integrated it into the concept of body image, which was a central issue in his entire scientific work. This article additionally addresses the question of whether Schilder’s assumptions are still valid today and what consequences might arise from this.

Der 1886 in Wien geborene Arzt und Wissenschaftler Paul Ferdinand Schilder (Abb. 1) hinterließ ein umfassendes und vielseitiges Werk. Beachtung fanden vor allem seine Beiträge auf psychoanalytischem und psychotherapeutischem Fachgebiet [2, 16, 26, 42, 46]. Zuletzt rückte die Bedeutung seines neurologischen Forschens in den Vordergrund. So gilt er als Erstbeschreiber der Encephalitis periaxialis diffusa, heute bekannt als die nach ihm benannte Schilder-Krankheit [19]. Doch das war nicht seine einzige neurowissenschaftliche Leistung. Dieser Artikel beleuchtet das Phänomen und die Entdeckungsgeschichte der Schmerzasymbolie – eines vielseitig interessanten und in der Medizin scheinbar fast vergessenen Störungsbilds, das Schilder ebenfalls als Erster beschrieben und abgegrenzt hat.

**Figure 1:**
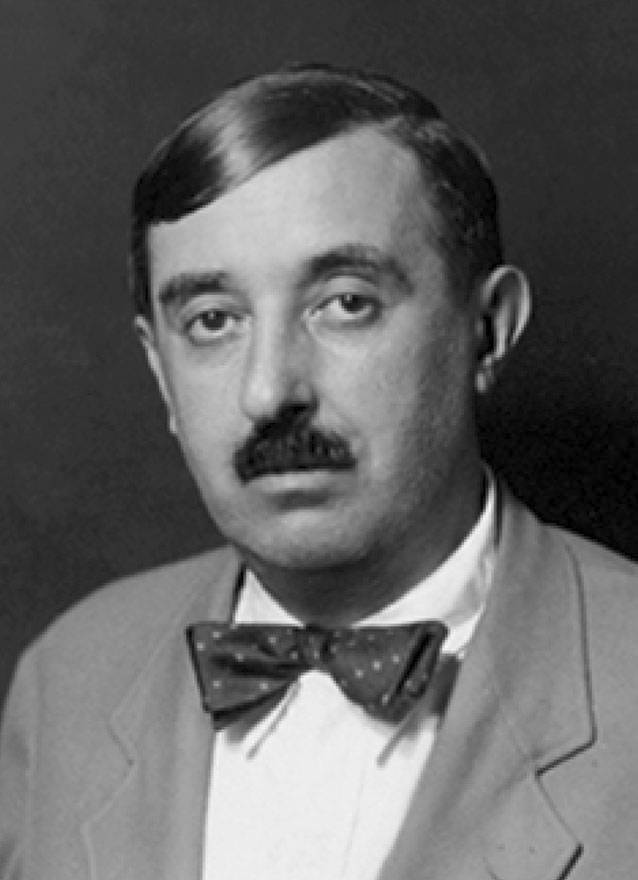

„Daddy, tut das weh?“ Es ist eine ungewöhnliche Frage, die der achtjährige Richard M. dem Vater da stellt, während er ihm seine blutende Hand zeigt. Doch der kleine Junge verspürt seit seiner Geburt keine Schmerzen. Er stand im Mittelpunkt eines Artikels im Nachrichtenmagazin *DER SPIEGEL* aus dem Jahr 1955, in welchem scheinbar erstmals eine breitere Öffentlichkeit von der sogenannten Schmerzasymbolie erfuhr [3] – einem Störungsbild, das der Arzt und Wissenschaftler Paul Ferdinand Schilder knapp drei Jahrzehnte zuvor beschrieben hatte.

Doch was ist Schmerzasymbolie? Bevor wir uns Schilders spannender Pionierarbeit widmen, soll dies kurz erklärt werden. Denn wahrscheinlich wird nicht jeder Mediziner mit dieser, heute als selten geltenden, Störung vertraut sein.

Bei der Schmerzasymbolie ist die somatische Schmerzwahrnehmung an und für sich nicht beeinträchtigt. Das heißt: Nozizeptoren, periphere Nervenleitung, Tractus spinothalamicus im Rückenmark, die Umschaltung im Thalamus und die Verarbeitung im somatosensiblen Kortex des Großhirns sind intakt. Für die affektiven Aspekte des Schmerzerlebens sind nun aber Verbindungen zum limbischen System verantwortlich [33]. Umschriebene Großhirnläsionen, insbesondere im Bereich der Inselrinde und der Projektionen zum Gyrus cinguli, können diese Verbindungen stören und so zu einer Schmerzasymbolie führen (Abb. 2). Dann fehlt die negative Konnotation und damit auch eine normale Reaktion auf den akuten Schmerz wie ein Abwehr‑/Schutzverhalten oder eine emotionale Beteiligung im Sinne eines Leidens. Die Folgen sind u. a. häufige und unbemerkte Selbstverletzungen – wie bei dem jungen Richard [8, 15, 35, 44].

**Figure 2:**
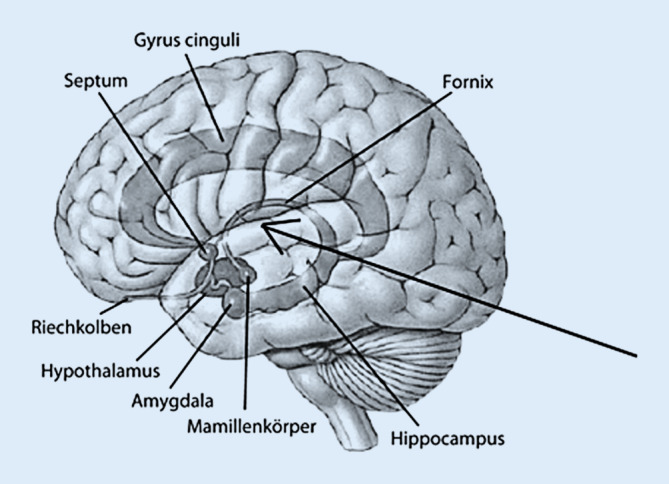

Doch viele Jahre zuvor war es eine ältere Dame, die Paul F. Schilder auf die Spur der Schmerzasymbolie brachte. Ihr Name war Anna H.

## Der Fall der Anna H.

### Ein gewöhnlicher Schlaganfall?

Sie steht im Mittelpunkt der frühesten bekannten Publikation zum Thema Schmerzasymbolie, veröffentlicht im Jahr 1928 von Paul F. Schilder und dessen damaligem Schüler Erwin Stengel (1902–1973; [38]).

Anna H. wurde am 23. August 1927 im Alter von 73 Jahren in die Psychiatrisch-Neurologische Klinik der Universität Wien eingeliefert. Der einweisende Arzt vermutete einen Schlaganfall. Die bis zu diesem Zeitpunkt gesunde Frau, die sogar noch als Verkäuferin gearbeitet hatte, war zu Hause „psychisch schwer verändert“ mit einer Sprach- und Verhaltensstörung vorgefunden worden [38, S. 143].

In der neurologischen Untersuchung fielen eine unflüssige Aphasie und eine Apraxie auf. Bis hierhin könnte man meinen, es handelte sich um einen zwar beeinträchtigenden, aber nicht außergewöhnlichen Schlaganfall in der linken Hirnhälfte. Dann jedoch entdeckten die behandelnden Ärzte etwas Merkwürdiges.

### Tut ihr gar nichts weh?

Auf Schmerzreize, deren Prüfung damals wie heute Bestandteil einer jeden neurologischen Untersuchung war, reagierte sie eigentümlich. Mal blieb sie völlig ruhig und unbeeindruckt, mal zog sie die malträtierte Extremität nur minimal zurück, dann wieder rieb sie sich bloß über eine soeben gestochene Hautstelle.

Anscheinend motiviert durch diese wundersame Entdeckung, probierten die Untersucher die verschiedensten Arten der Schmerzapplikation aus: Nadelstiche, Schlagen, Kneifen. Sie testeten dies an Armen, Beinen, am Bauch, im Gesicht und an der Zunge – also im Grunde genommen am gesamten Körper der Anna H. Auch Übergießen der Extremitäten mit kaltem Wasser, starke Hitzereize (offene Flamme am Finger) sowie schrilles Pfeifen direkt neben dem Ohr und das blendende Abbrennen eines Magnesiumdrahts direkt vor den Augen schienen der Patientin kein Unbehagen zu bereiten.

Allerdings fiel auf, dass all diese Reize sehr wohl von ihr bemerkt wurden, keinesfalls wirkte sie gleichgültig oder unaufmerksam. Ganz im Gegenteil! Sie wendete sich den Untersuchern und Schmerzreizen teils aktiv zu, wirkte interessiert, bot sich und ihren Körper gar bereitwillig an. So präsentierte sie zum Beispiel nach Heben ihres Rocks eine zuvor gestochene Stelle am Oberschenkel und sagte: „Da können Sie!“ [38, S. 146]. Oft rieb sie sich das soeben „geprüfte“ Areal, als ob es jucken würde, manchmal lachte sie, wirkte kitzelig. Ihr Gesichtsausdruck zeigte durchaus Veränderungen wie bei normalem Schmerzempfinden. Gleichwohl registrierten die Untersucher das Fehlen typischer vegetativer Reaktionen wie Veränderungen der Pupillenweite oder Herzfrequenz. Anna H. bejahte die Frage nach Schmerzen, antwortete darauf mit paradox anmutenden Sätzen wie „Au weh, das hat gutgetan!“ oder „Danke schön, das war ganz gut, das hat mir so weh getan!“ [38, S. 147]. Nach besonders starken Schmerzreizen zuckte sie zwar kurz mit dem Arm oder Bein. Zu keinem Zeitpunkt reagierte sie aber auf eine eigentlich zu erwartende Weise, nämlich mit Wegziehen einer Extremität, Abwenden ihres Körpers oder dem Versuch, der – objektiv betrachtet – quälenden Situation irgendwie zu entfliehen. Auch blieb sie den Untersuchern gegenüber stets freundlich und höflich, witterte offensichtlich keine Gefahr in ihnen. Selbst nicht, als diese eindeutig bedrohliche Gesten mit u. a. Messer und Hammer ausführten. Sie ging sogar noch weiter, fügte sich mit Gegenständen wie Nadel und Pinzette selbst Verletzungen zu. Dabei wirkte sie neugierig und geradezu fasziniert.

### Einordnung und Abgrenzung

Ob Schilder selbst zu den Untersuchern der Anna H. gehörte, ist nicht sicher überliefert. Allerdings liegt dies nahe, schließlich war er Erstautor der Fallbeschreibung und zu dieser Zeit Arzt an der Psychiatrisch-Neurologischen Universitätsklinik Wien. Diese stand damals unter der Leitung von Julius Wagner-Jauregg (1857–1940), einem innovativ denkenden, forschenden und später mit dem Nobelpreis bedachten Nervenarzt [18, 45]. Schilder selbst war an die ihn ausbildende Universität seiner Heimat zurückgekehrt, nachdem er Station bei Gabriel Anton (1858–1933) in Halle und Paul Flechsig (1882–1920) in Leipzig gemacht hatte. Sein Wirken in Mitteldeutschland war insbesondere bedeutsam, da er sich in dieser Zeit verdient gemacht hatte um die Erstbeschreibung und Definition der Encephalitis periaxialis diffusa, heute besser bekannt als Schilder-Krankheit [19]. Im Jahr 1920 hatte sich Schilder für Neurologie und Psychiatrie habilitiert, seit 1925 lehrte und forschte er als Professor in Wien [26, 46].

Welche Schlüsse zog Schilder nun aus der Krankengeschichte der Anna H.? Zunächst einmal ging auch er von einem Schlaganfall als Ursache der akut aufgetretenen Symptomatik aus. Die Aphasie hielt an, die Apraxie war recht rasch rückläufig. Dazu gesellte sich nun die Störung der Schmerzwahrnehmung. Die in leichter Ausprägung bemerkte „Einstellungsstörung“ [38, S. 153] – dieser heute nicht mehr gebräuchliche Begriff steht für die Schwierigkeit, aus einem bestimmten Gedankenkreis herauszukommen und die Aufmerksamkeit auf andere Inhalte zu lenken [29] – könnte, so Schilder, nicht verantwortlich sein. Denn auch in Situationen, in welchen sich Anna H. sicher auf den Schmerzreiz konzentrierte, sich ihm teilweise ja sogar direkt zuwandte und manchmal selbst zufügte, zeigte sie keine normale, aversive Reaktion. Auch habe sich die Einstellungsstörung zurückgebildet, während die Schmerzunempfindlichkeit angehalten habe. Eine Sensibilitätsstörung durch den Schlaganfall scheide als Ursache aus, schließlich sei der gesamte Körper betroffen. Zudem bemerkte Anna H. den Schmerz ja ganz offensichtlich – es lag also keine Analgesie vor – was Schilder zurückführte auf die durchaus vorhandenen Reaktionen wie angedeutete Abwehrbewegungen, Reaktionen in Sprache und Mimik sowie das Reiben betroffener Hautstellen. Es schien allerdings so, als ob die Patientin den Schmerzreiz unbewusst falsch eingeschätzt bzw. eingeordnet habe. So verhielt sie sich dem Untersucher gegenüber freundlich zugewandt, egal wie heftig die Reize wurden. Für Schilder wirkte es, als sei das „Schmerzerlebnis beiseitegeschoben“ im Sinne einer „Verdrängung im Bereiche des Organischen“ [38, S. 154]. Doch nicht nur das Schmerzerleben, sondern sogar das Gefühl für eine Bedrohung im Allgemeinen schien zu fehlen. Schilder folgerte, die Störung betreffe nicht das „Rohmaterial der Wahrnehmung … sondern die höheren Stufen des Wahrnehmungsaktes“ [38, S. 154], beeinträchtigt sei also „der Aufbau der Schmerzwahrnehmung“ [38, S. 155].

Schilder stellte fest, vergleichbare Fälle seien bisher in der Fachliteratur nicht berichtet worden. Er schlug für das Störungsbild den Begriff „Schmerzasymbolie“ vor und sah einen Zusammenhang mit „den übrigen Asymbolien“ [38, S. 155]. Der Begriff Asymbolie, eingeführt von dem Nervenarzt und Sozialhygieniker Carl Maria Finkelnburg (1832–1896), wurde in der Geschichte der Nervenheilkunde unterschiedlich definiert bzw. gebraucht und ist heute aus der Mode gekommen [1, 32]. Man darf in diesem Kontext Asymbolie im Sinne einer Agnosie, also einer Erkennungsstörung, verstehen.

Es war Schilder wichtig, die Schmerzasymbolie abzugrenzen von Zuständen reduzierter bzw. aufgehobener Schmerzempfindung, wie man sie bei verschiedenen psychischen Erkrankungen wie katatonen Psychosen, der Hysterie oder der Manie beobachtet hatte und auch noch heute kennt [12]. Verantwortlich machte Schilder bei diesen Krankheitsbildern eine „Vernachlässigung der Schmerzwahrnehmung durch die Persönlichkeit“ [38, S. 155], sozusagen eine mangelnde Aufmerksamkeit gegenüber dem Schmerz. Er geht an dieser Stelle zwar nicht direkt darauf ein, doch wird er wohl an die Erkenntnisse von Paul Eugen Bleuler (1857–1939) gedacht haben, dem bekannten Züricher Psychiater, der sich vor allem auf dem Feld der Schizophrenieforschung verdient gemacht und bei Schizophreniepatienten Auffälligkeiten der Schmerzverarbeitung beschrieben hatte [9].

**Figure 3:**
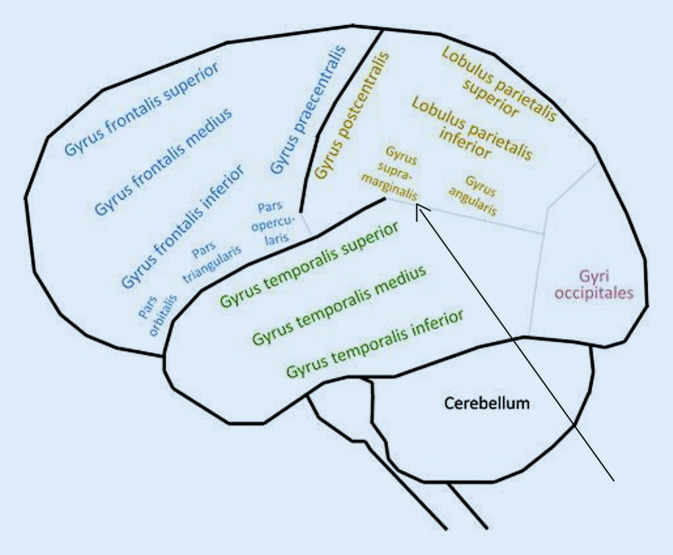

Nach ausführlicher Darstellung des Falls der Anna H. reichte Schilder in derselben Publikation nun gleich noch die kurze Beschreibung einer sehr ähnlichen, ebenfalls aktuellen Krankengeschichte nach. Auch hier ging es um eine Patientin mit Aphasie und Apraxie, die eine Störung des Schmerzempfindens zeigte. Schilder folgerte daraus, die Schmerzasymbolie sei kein seltenes Phänomen. Bisher habe man sie wohl übersehen oder als Resultat einer Aufmerksamkeitsstörung interpretiert. Schilder schloss seinen Aufsatz mit Überlegungen zum Ort der verantwortlichen Hirnläsion ab. Er ging nicht von einer „allgemeinen Hirnschädigung“ aus, sondern postulierte ein „Lokalsymptom“ [38, S. 156] analog zur Aphasie. Anhand der klinischen Beschwerden und aufgrund des Fehlens bestimmter Symptome wie z. B. einer Sehstörung grenzte Schilder die Lokalisation ein auf den Bereich des linken Gyrus supramarginalis im Lobulus parietalis inferior, also den vorderen Anteil des unteren Scheitel‑/Parietallappens (Abb. 3). Schilder betonte, dies bedeute keinesfalls, dort sei gezwungenermaßen die Funktion für ein normales Schmerzerleben lokalisiert. Es seien wohl weitere Hirnareale bzw. die Verbindungen dorthin wichtig, um eine „höhere Integration“ [38, S. 157] zu gewährleisten.

Wie ging es indes mit der Patientin Anna H. weiter? Sie lebte nach ihrem Schlaganfall noch drei Monate. In dieser Zeit hielt die Aphasie an, der Zustand verschlechterte sich fortschreitend und letztlich starb sie infolge kardialer Dekompensation. Jetzt war es möglich, ihr Gehirn makro- und mikroskopisch zu untersuchen – Jahrzehnte vor der ersten Anwendung der Computer- oder Magnetresonanztomographie die einzige Chance, die Annahmen über die zugrunde liegende Pathologie zu überprüfen.

### Ein Blick ins Hirn

Die Ergebnisse der Obduktion machte Schilder in seiner Publikation „Der Hirnbefund bei Schmerzasymbolie“ [39] noch im Jahre 1928 der Öffentlichkeit zugänglich.

**Figure 4:**
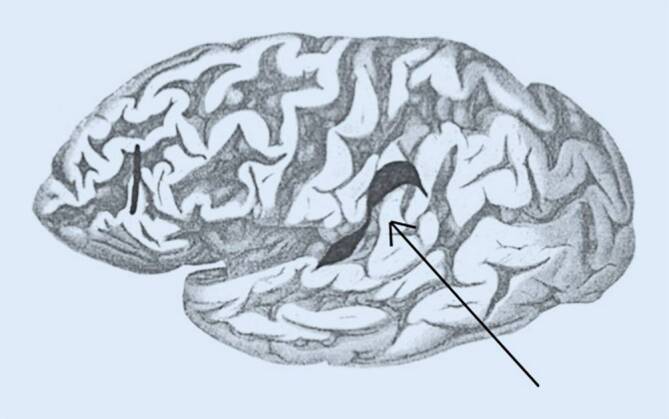

Von außen war das Gehirn bis auf eine frontal und okzipital betonte Atrophie unauffällig. Nach Formalinfixierung und Zerlegung in Scheiben folgte nun die systematische Untersuchung der makroskopischen Veränderungen. Dabei fand sich ein älterer Herd im linken Frontallappen, dem Schilder unter Berücksichtigung der Lokalisation keine Bedeutung für die Symptomatik beimaß. Als richtungsweisend hingegen wertete er eine zweite Läsion im linken Parietallappen unter Miteinbeziehung der Wernicke-Region und des unteren Teils des Gyrus supramarginalis (Abb. 4). Die nach dem deutschen Neurologen Carl Wernicke (1848–1905) benannte Region bildet das sensorische Sprachzentrum, eine dort lokalisierte Schädigung erklärt somit die Störung der Flüssigkeit im sprachlichen Ausdruck der Patientin. Schilder hatte in seiner vorangegangenen Publikation als wahrscheinlichen Läsionsort für die Schmerzasymbolie den Gyrus supramarginalis angenommen. Darin konnte er sich nun bestätigt sehen. Als gerechtfertigt erachtete er auch den für die Störung gewählten Terminus. Hatte er in seiner vorangegangenen Publikation den Begriff Schmerzasymbolie lediglich vorgeschlagen, verwendete er ihn jetzt selbstbewusst. Und es war ihm wichtig, nochmals zu betonen, es handele sich „um eine Störung im Aufbau der höheren Komponenten der Schmerzwahrnehmung“ [39, S. 537]. Diesen Gedanken entwickelte er weiter und setzte ihn folgend in Beziehung zum Konzept des „Körperschemas“, welches ihn zeitlebens beschäftigte.

### Detaillierte Untersuchung zwei Jahre später

Was noch fehlte, war die Mitteilung der mikroskopischen Befunde. Diese reichte Schilder zwei Jahre später nach, in seinem Aufsatz „Das Krankheitsbild der Schmerzasymbolie“ [40]. Mit der für ihn typischen Akribie und Sorgfalt beschrieb er die Beschaffenheit und Ausdehnung der beiden bereits erwähnten Herde. Hierzu fertigte er Schnittserien an und verwendete verschiedene Färbungen. So fand er in den Herden Fettkörnchenzellen (= Makrophagen im Zentralnervensystem), Gliawucherungen und Vermehrungen der Gefäße sowie des Bindegewebes – insgesamt also einen „für eine Erweichung typischen Befund“ [40, S. 253]. Unter einer „Erweichung“ – heute wird der Begriff kaum mehr verwendet – versteht man in der Neuropathologie die Kolliquationsnekrose nach einem ischämischen Hirninfarkt [30]. Nun war Anna H.s klinisch gestellte Diagnose histologisch bestätigt.

## Etwas kristallisiert sich heraus …

**Figure 5:**
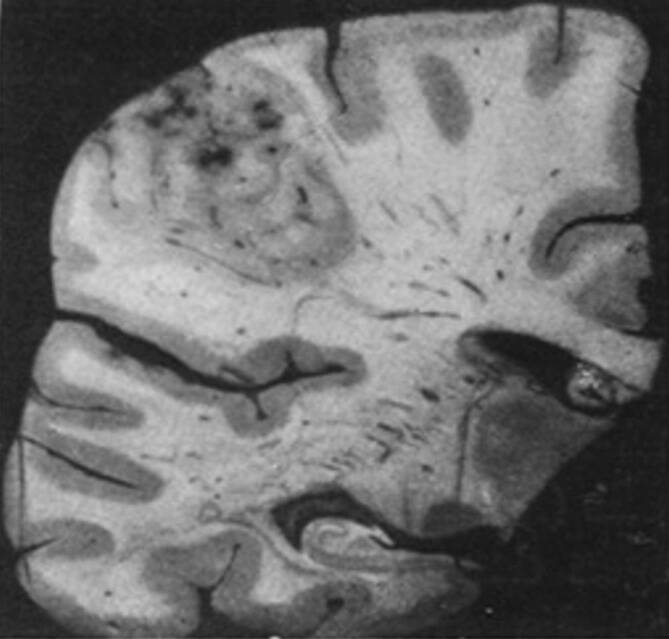

Um seine Hypothese zur Lokalisation struktureller Veränderungen bei Patienten mit Schmerzasymbolie zu untermauern, präsentierte Schilder ausführlich einen weiteren, nunmehr dritten Fall aus der Wiener Universitätsklinik [40]. Eine 63-jährige Patientin hatte u. a. eine Aphasie, eine Hemiparese rechts und eine Apraxie entwickelt. Auffällig war wieder eine fehlende normale Reaktion auf Schmerzreize. Nach zwei Monaten starb sie. In der Obduktion zeigten sich zwei Hirntumoren, genauer gesagt Gliome. Einer im linken Frontalhirn und der andere im linken Parietallappen unter Miteinbeziehung des Gyrus supramarginalis (Abb. 5). Eine dortige Läsion als Ursache der Schmerzasymbolie hatte Schilder beim Fall der Anna H. bereits klinisch vermutet und in der Obduktion bestätigt gefunden. Mittels differenzierter Überlegungen zu Lokalisation und Funktion stellte Schilder nun dar, warum auch in diesem Fall aus seiner Sicht die Störung der Schmerzverarbeitung am ehesten begründet sei in einer Läsion des Gyrus supramarginalis und nicht des Frontallappens. Nicht nur um dem nachzugehen, präsentierte er einige weitere ähnliche Krankheitsgeschichten.

## Noch mehr Schmerzasymboliker werden entdeckt

Zunächst berichtete der Wiener Professor über eine 44-jährige Patientin, die an seiner Klinik aufgenommen worden war. Nach Entfernung eines Tumors im linken Parietallappen – eine Schädigung u. a. des Gyrus supramarginalis wurde angenommen – entwickelte sie den mittlerweile bekannten Symptomkomplex aus Aphasie, Apraxie und Schmerzasymbolie. Ähnlich wie Anna H. reagierte sie auf Schmerzreize merkwürdig. Nachdem man in einen ihrer Finger stach, beklagte sie verbal zwar Schmerzen, lächelte aber und bot ihre anderen Finger der Untersuchernadel freundlich an. Auch akustische oder optische Bedrohungen beeindruckten sie nicht. Schilder fand, ihr Verhalten sei „den Ambivalenzreaktionen von Neurotikern und Geisteskranken“ ähnlich, er fühlte sich an die „Affektdissoziation Schizophrener“ erinnert. Man müsse also davon ausgehen, dass bei Menschen mit Schmerzasymbolie „das Schmerzerlebnis irgendwie abgeändert ist, sei es, dass es ambivalent, sei es, dass es einfach paradox, d. h. lustbetont erlebt wird“ [40, S. 257]. Auf eine allgemeine Aufmerksamkeitsstörung wie bei Patienten mit sensorischer Aphasie, wie sie von Ludwig Lichtheim (1845–1928) und Wernicke beschrieben worden war, könne man die Schmerzasymbolie auch in diesem Fall nicht zurückführen.

Dieser Krankengeschichte ließen Schilder und der nach wie vor mit ihm forschende Stengel die Darstellung sieben weiterer Kasuistiken folgen [40]. Die jeweiligen Patienten wurden offensichtlich alle zwischen 1928 und 1930 untersucht bzw. behandelt. Manche in der Wiener Universitätsklinik, andere im New Yorker Bellevue Hospital, wo Schilder seit 1929 Direktor der Psychiatrischen Abteilung war. Heterogene Hirnschädigungen spielten eine Rolle: Infektion (Neurolues), ischämischer Schlaganfall, Hirnblutung, Tumor, Schädeltrauma. Eine Obduktion war nicht immer möglich, stets jedoch eine eingehende klinische Untersuchung, Beobachtung und differenzialdiagnostische Einschätzung. Alle Betroffenen zeigten die bereits beschriebenen Auffälligkeiten im Schmerzerleben. Es gab aber Unterschiede. So bildete sich die Störung des Schmerzerlebens in manchen Fällen zurück, teils bestand eine Differenz der Ausprägungsgrade an oberen und unteren Extremitäten. Meistens lagen eine Aphasie und eine Apraxie vor, mitunter aber kein Zusammenhang zwischen einer Dynamik dieser Symptome und der Schmerzasymbolie.

## Schilders Fazit und Ausblick

In der Summe blickte Schilder nun auf elf ausführlich klinisch untersuchte, im Verlauf beobachtete und wenigstens teilweise histopathologisch aufgearbeitete Fälle zurück, die er seiner Schmerzasymbolie zurechnete. Für ihn der Anlass, ein Fazit zu ziehen. So kam er zu der Überzeugung, die Schmerzasymbolie sei eine „typische und häufige“ Störung [40, S. 275] – wenngleich er einräumte, dass ihm, sicherlich begründet in seiner Tätigkeit an zwei großen Nervenkliniken mit ausgedehnter Patientenversorgung, außerordentlich viele Krankengeschichten zugänglich gewesen seien. Gedanken machte er sich darüber, warum niemand vor ihm dieses Syndrom beschrieben hatte. Seine Antwort: Man habe die veränderte Schmerzreaktion wohl zurückgeführt auf eine Aufmerksamkeitsstörung. Zudem könne die, meist begleitende, Sprachstörung zu einem Übersehen beigetragen haben. Da bei den Betroffenen die Einstellung nicht nur dem Schmerz, sondern allgemein potenziellen Gefahren gegenüber gestört war, dürfe man sogar den Begriff „Gefahrasymbolie“ verwenden. Schilder zog es jedoch vor, den ursprünglich gewählten Namen beizubehalten. Letztlich zählte er die Schmerzasymbolie zu den „Störungen im Bereich des Körperschemas im weitesten Sinne“ [40, S. 276]. Aus der Vielfalt der zugrunde liegenden Hirnschädigungen schloss Schilder, es komme bei der Schmerzasymbolie auf den Ort der Läsion an und nicht auf deren Ursache. Er thematisierte jetzt auch nochmals eigene frühere Überlegungen zum Läsionsort. So glaube er weiterhin, der Gyrus supramarginalis spiele eine Rolle. Jedoch solle dies künftig noch ausführlicher untersucht werden. In jedem Fall ging er von einer kortikalen, d. h. die Großhirnrinde betreffenden, Parietallappenschädigung aus und nicht von einer Marklagerläsion. Nachgehen müsse man auch dem „paradoxen Verhalten“ [40, S. 277] einiger Patienten, die sich dem Schmerz mitunter aktiv zugewendet, ihn sich sogar selbst zugefügt hatten.

Ein Jahr später, also 1931, fasste Schilder seine Beobachtungen und Überlegungen zusammen in einem kleinen englischen Artikel, publiziert in einer großen amerikanischen Fachzeitschrift [41]. Den Begriff Schmerzasymbolie übersetzte er dabei mit „asymbolia for pain“. Ziel war es sicherlich, eine größere Leserschaft zu erreichen. Außerdem hatte Schilder, wie bereits erwähnt, nun in New York seinen Lebens- und Schaffensmittelpunkt. Dort blieb er auch – bis zu seinem frühen Unfalltod im Jahr 1940, als er von einem Auto überfahren wurde [16, 26].

## Eine „typisch Schildersche“ Leistung

Paul F. Schilders wissenschaftlich-ärztliche Arbeitsweise auf diesem Gebiet weist viele Parallelen zu der von ihm ca. 15 Jahre zuvor geleisteten Erstbeschreibung und Abgrenzung der Encephalitis periaxialis diffusa auf [19]. Grundlage seiner Überlegungen war stets eine ausführliche und detaillierte klinische Beschreibung, die er dann zusammen betrachtete mit den Ergebnissen einer, wenn möglich selbst durchgeführten, eingehenden histopathologischen Aufarbeitung des Hirngewebes. Die so gewonnenen Erkenntnisse führten zu Annahmen bzw. Hypothesen, die er selbstbewusst weiterentwickelte. Dabei betrieb er großen Aufwand, befasste sich akribisch mit einer Vielzahl von Patienten und setzte sich kritisch mit der Fachliteratur auseinander. Heuristisch vorgehend scheute er sich nicht, eigene Ansichten zu hinterfragen oder Grenzen in der Beurteilbarkeit mancher Sachverhalte einzuräumen.

In den Veröffentlichungen zur Schmerzasymbolie tritt immer wieder Schilders Interesse auch an geisteswissenschaftlichen und neuropsychologischen Fragestellungen zutage. Dies ist verständlich, schließlich hatte er neben Medizin auch Psychologie und Philosophie studiert.

Vielleicht rechnete er auch deshalb die neu entdeckte Schmerzasymbolie den Agnosien zu? Agnostizismus als Weltanschauung, der Agnostiker als „Unwissender“ im Hinblick auf die Existenz Gottes oder als „Nichterkennender“ in der Philosophie sind geläufige Begriffe. Der medizinisch-neurowissenschaftliche Terminus leitet sich etymologisch von derselben altgriechischen Wortwurzel ab. Man versteht darunter eine beeinträchtigte Interpretation bestimmter Wahrnehmungen. Sinnesorgane, afferente Reizleitung, primäre somatosensible, visuelle oder auditorische Hirnrinde sind dabei intakt, Vigilanz und Konzentrationsfähigkeit gegeben. Ein- oder beidseitige Hirnläsionen im Bereich des Parietal‑, Okzipital- oder Temporallappens verhindern jedoch durch eine sekundäre Verarbeitungsstörung ein normales Erkennen. Agnosien wurden beschrieben für alle möglichen Sinnesmodalitäten. Hervorzuheben ist die visuelle Agnosie mit Unterformen wie z. B. Prosopagnosie (gestörtes Erkennen von Gesichtern), Achromatopsie (Farbsehstörung) und Simultanagnosie (mehrere gleichzeitig dargebotene Objekte werden nicht erkannt). Daneben gibt es u. a. die Astereognosie (Gegenstände können nicht ertastet werden), Alexie (Lesestörung) und akustische Agnosie. Eine Sonderstellung nimmt die Asomatognosie ein, bei der ein Körperteil als nicht zum eigenen Körper gehörend betrachtet wird. Sie steht damit in enger Beziehung zur Anosognosie, welche charakterisiert ist durch eine fehlende oder beeinträchtigte Wahrnehmung neurologischer Störungen. Diese kann z. B. eine Lähmungserscheinung betreffen oder eine kortikale Blindheit. Wird Letztere nicht selbst erkannt, spricht man von einem Anton-Syndrom [24, 25]. Insgesamt sind Agnosien eher selten, haben für die Betroffenen aber oft schwerwiegende Alltagsfolgen [6, 20].

Eingeführt wurde der Begriff der Agnosie Ende des 19. Jahrhunderts von Sigmund Freud (1856–1939). Später trug Hugo Liepmann (1863–1925), selbst ein Schüler Wernickes und bekannt vor allem für seine Arbeiten zu den Apraxien, wesentlich zum Wissensstand bei [27]. Vergegenwärtigt man sich die Gemeinsamkeiten der Agnosien und der Schmerzasymbolie, kann man Schilder auch aus heutiger Sicht zustimmen und die Schmerzasymbolie als Agnosie begreifen.

Die Verbindung zum Konzept des Körperschemas wurde bereits erwähnt. Laut Schilder handelt es sich dabei um „das Wissen vom eigenen Körper, das Bild, das wir vom eigenen Körper in uns tragen“ [39, S. 537]. Dieses Konzept war von Arnold Pick (1851–1924) erstbeschrieben worden [32], wichtige Beiträge stammen von Henry Head (1861–1940; [1, 17, 31]). Auch Schilder hatte sich eingehend mit diesem Thema beschäftigt, es besaß im Grunde fast während seiner gesamten wissenschaftlichen Laufbahn einen zentralen Stellenwert [26]. Seine Auseinandersetzung mit dem Körperschema demonstriert beispielhaft, wie er gleichermaßen als naturwissenschaftlicher Neurologe und als psychologisch orientierter Psychiater dachte bzw. forschte. Die Schmerzasymbolie interpretierte er als „Dissoziation zwischen dem sonst intakten Körperschema und der Schmerzwahrnehmung“ [39, S. 537], somit vollzog er eine Integration seines neu entdeckten Störungsbilds in das Konzept des Körperschemas.

Methodisch stehen Schilders Publikationen zur Schmerzasymbolie exemplarisch für das zeitgenössische Vorgehen in der Neurologie und den Neurowissenschaften. Seine Fokussierung auf die Zusammenhänge zwischen gestörter Funktion und Lokalisation der verantwortlichen Läsion teilten bekannte Pioniere der Neurologie wie z. B. Pierre Paul Broca (1824–1880; Arbeiten zur Aphasie), Gabriel Anton (1858–1933; Beschreibung der visuellen Anosognosie) und Joseph Babinski (1857–1932; Untersuchung von Pyramidenbahnläsionen; [7, 13, 24, 36, 43]).

## Schmerzasymbolie heute

Zunächst nochmals kurz zurück zu dem anfangs erwähnten achtjährigen Richard M. Litt er wirklich unter einer Schmerzasymbolie? Erstaunlicherweise handelte es sich in keinem der Schilderschen Fälle um eine angeborene Hirnschädigung. Kinder waren nie betroffen, sondern stets Erwachsene im eher fortgeschrittenen Alter. Wahrscheinlich lag bei Richard eher eine Analgesie vor, möglicherweise als Resultat einer Neuropathie oder eines Ionenkanaldefekts [11, 34]. Der *SPIEGEL* hat sich hier vermutlich geirrt.

Wie sind indes heutzutage Schilders Ausführungen zur Schmerzasymbolie zu beurteilen? Als Erstes fällt eine Diskrepanz zwischen der von ihm postulierten und von uns aktuell wahrgenommenen Prävalenz auf. Wir erinnern uns – er vermutete eine „typische und häufige“ Störung [40, S. 275], hatte innerhalb von nur zwei Jahren bei elf Patienten Schmerzasymbolie diagnostiziert. „Typisch“ meint Schilder hier vermutlich im Sinne von „charakteristisch“ für Patienten mit Hirnschädigungen. Wirft man einen Blick in heutige Standardlehrbücher der Neurologie und Neuropsychologie, findet man dazu fast nichts. Die ausführliche, internetbasierte Literaturrecherche u. a. in PubMed liefert relativ wenige Artikel aus den letzten Jahrzehnten, die dann auch oft Einzelfallberichte oder eher philosophischer Natur sind [4, 14, 22, 29]. Wir wagen sogar zu behaupten, die meisten Leser dieses Artikels haben zuvor noch nie etwas von der Schmerzasymbolie gehört. Hat sich Schilder also geirrt und vielleicht ein inhomogenes Patientenklientel mit ganz unterschiedlichen Ursachen der Schmerzwahrnehmungsstörung präsentiert? Deren gibt es schließlich viele (Tab. 1). Wenig vorstellbar, wenn man sich seine wissenschaftliche Genauigkeit und Differenziertheit vor Augen führt. Zudem ist die Schmerzasymbolie als Störungsbild wissenschaftlich anerkannt und der Wissensstand hat sich seit der Veröffentlichung von Schilders Aufsätzen nur punktuell erweitert. So wurde in den folgenden Jahrzehnten die Schmerzasymbolie von manchen Autoren im Zusammenhang mit Diskonnektionssyndromen gesehen [8, 28]. Und mithilfe moderner Schnittbildgebungen ist es freilich gelungen, die maßgeblichen Läsionsorte noch besser zu definieren. Man weiß jetzt um die Bedeutung der Inselrinde und der Projektionen zum Gyrus cinguli [10, 15, 35, 44]. Der Gyrus supramarginalis allein scheint nicht die entscheidende Rolle zu spielen, wohl aber die entsprechende Hirnregion.

**Table 1:** 

Ursache	Betroffene Körperareale	Besonderheiten
Läsion peripherer Nerven oder Hirnnerven	Entsprechendes Versorgungsgebiet	Initial oft mit Schmerzen, mit Hypästhesie, je nach Qualität des Nervs auch motorische oder autonome Ausfälle
Plexusschädigung	Versorgungsgebiet mehrerer peripherer Nerven bzw. Segmente	Immer mit weiteren Ausfällen, initial und oft auch chronisch mit Schmerzen
Dissoziierte Sensibilitätsstörung	Kontralateral zu einer Läsion im Tractus spinothalamicus oder Thalamus	Auch Temperaturempfindung beeinträchtigt, Oberflächensensibilität intakt, oft verbunden mit Missempfindungen, häufig Folge von Tumor oder Entzündung
Polyneuropathie	Initial meist distale (untere) Extremitäten	Häufig, viele mögliche Ursachen (insbesondere Diabetes mellitus und Alkoholabusus), meist weitere Sensibilitätsstörungen und Symptome
Schädigung des Gyrus postcentralis (primär sensible Hirnrinde)	Kontralaterale Körperseite	Isolierte Schmerzwahrnehmungsstörung sehr unwahrscheinlich, weitere Symptome
Schmerzasymbolie	Gesamter Körper	Algesie erhalten, aber keine negative Konnotation/kein Leiden aufgrund gestörter Verbindung zum limbischen System
Psychogene Störung	Variabel	Bei psychischen Erkrankungen wie z. B. Schizophrenie, dissoziativen Episoden; stets weitere Symptome
*Kongenitale Analgesie*		
Mutationen in bestimmten Natriumkanälen (Gen *SCN9A* und *SCN11A*), „congenital insensitivity to pain“ (CIP)	Jeweils gesamter Körper	Sehr selten, Sensibilität sonst intakt, oft Riechstörung
Hereditäre sensible und autonome Neuropathie (HSAN) Typ III = familiäre Dysautonomie (Riley-Day-Syndrom)		Weitere Symptome, fast ausschließlich osteuropäische Juden sind betroffen (Grund hierfür ist unklar)
HSAN Typ IV = „congenital insensitivity to pain with anhidrosis“ (CIPA)		Weitere Symptome, extrem selten

Hat Schilder die Häufigkeit überschätzt, im Eifer nur gesehen, was er auch sehen wollte? Möglich, denn er war von Anna H.s Krankengeschichte gleichermaßen beeindruckt und verwundert, man spürt förmlich den geweckten Forschergeist bei der Lektüre seiner Originalarbeiten. Unterlagen seine Untersuchungen ähnlicher Patienten in den folgenden Jahren also evtl. einem Selektionsbias, wie man das heute nennen würde? Das wäre denkbar. Oder aber wir Ärzte bemerken die Störung einfach nicht, weil uns die Kenntnis darüber fehlt – so, wie es Schilder seinen Zeitgenossen unterstellt hat. Die Erfahrung, dass Menschen sehr variabel auf vergleichbare Schmerzauslöser reagieren – wir haben sie alle schon gemacht – führen wir vielleicht manchmal zu leichtfertig zurück auf individuelle körperliche, mit dem Geschlecht verknüpfte, psychische oder kulturell-religiöse Faktoren. Grundsätzlich ist das natürlich richtig und wichtig, seit der Antike sind Schmerzkonzepte und -erklärungsmodelle einem stetigen Wandel unterworfen und die zentralnervöse Schmerzmodulierung, auch im Zusammenhang mit den o. g. Faktoren, wird seit Jahrzehnten erforscht [37]. Doch könnte nicht hinter dem einen oder anderen Patienten mit auffällig reduziertem Schmerzempfinden ein „Schmerzasymboliker“ stecken?

Zwangsläufig stellt sich die Frage, ob man aus dem Vorliegen einer Schmerzasymbolie praktische Konsequenzen ableiten könnte. Dies wäre der Fall, wenn man sich die Möglichkeit einer „inkompletten“ Schmerzasymbolie ausmalt ohne Dichotomisierung zwischen normalem und aufgehobenem Schmerzempfinden – also das, was wir in der Regel mit Analgetika und nichtmedikamentöser Schmerztherapie erreichen wollen. Neuere Entwicklungen gehen bereits durchaus in diese Richtung, betrachtet man beispielsweise das noch recht junge Einsatzgebiet der tiefen Hirnstimulation bei chronischem Schmerz oder die Erkenntnis, dass erhöhte Konzentrationen endogener Cannabinoide – zumindest in der Inselrinde von Ratten – eine Art schmerzasymbolische Effekte verursachen [21].

Was auch immer uns in der Schmerzmedizin noch erwartet – Paul F. Schilder hat mit der Definition und Abgrenzung der Schmerzasymbolie vor 90 Jahren einen interessanten und spannenden Beitrag geleistet, der gewürdigt werden sollte und vielleicht noch Potenzial in sich birgt.

## Fazit für die Praxis

- Die Schmerzasymbolie (engl. „pain asymbolia“ oder „asymbolia for pain“) stellt ein heutzutage wenig bekanntes Störungsbild dar. Das Schmerzempfinden, d. h. die negative emotionale bzw. affektive Komponente des Schmerzerlebens, ist dabei aufgehoben. Dagegen sind Schmerzentstehung, Schmerzleitung und primäre Schmerzverarbeitung im Gehirn intakt. Verantwortlich sind Läsionen im limbischen System bzw. in assoziierten Arealen. Besondere Bedeutung scheinen hierbei die Inselrinde und der Gyrus cinguli zu haben.
- Als Erstbeschreiber gilt der Arzt und Wissenschaftler Paul Ferdinand Schilder (1886–1940), der zwischen 1928 und 1930 elf Fälle detailliert klinisch erfasst und einige davon ausführlich pathologisch untersucht hat.
- Möglicherweise kommt die Schmerzasymbolie häufiger vor, als heute angenommen. Die weitere Erforschung des Störungsbilds könnte u. a. lohnend sein, um Mechanismen der zentralen Schmerzverarbeitung besser verstehen und hiervon ggf. auch therapeutische Maßnahmen ableiten zu können.
