# Erratum zu: Die Schmerzasymbolie – um 1930 von Paul F. Schilder entdeckt und heute fast vergessen?

**DOI:** 10.1007/s00482-021-00609-7

**Published:** 2021-11-26

**Authors:** Martin Jahn, Holger Steinberg

**Affiliations:** 1 Forschungsstelle für die Geschichte der Psychiatrie, Klinik und Poliklinik für Psychiatrie und Psychotherapie, Medizinische Fakultät, Universität Leipzig, Semmelweisstraße 10, 04103 Leipzig, Deutschland; 2 Neurologisches Rehabilitationszentrum Leipzig, Bennewitz, Deutschland

**Erratum zu:**

**Der Schmerz 2020**

10.1007/s00482-020-00447-z

Der Artikel „Die Schmerzasymbolie – um 1930 von Paul F. Schilder entdeckt und heute fast vergessen?“ von Martin Jahn und Holger Steinberg wurde ursprünglich Online First ohne „Open Access“ auf der Internetplattform des Verlags publiziert. Nach der Veröffentlichung in Band 34, Heft 2, pp. 172–180 hatten sich die Autoren für eine Open-Access-Veröffentlichung entschieden. Das Urheberrecht des Artikels wurde deshalb in „© Der/die Autor(en) 2020“ geändert. Dieser Artikel ist jetzt unter der Creative Commons Namensnennung 4.0 International Lizenz veröffentlicht, welche die Nutzung, Vervielfältigung, Bearbeitung, Verbreitung und Wiedergabe in jeglichem Medium und Format erlaubt, sofern Sie den/die ursprünglichen Autor(en) und die Quelle ordnungsgemäß nennen, einen Link zur Creative Commons Lizenz beifügen und angeben, ob Änderungen vorgenommen wurden.

Die in diesem Artikel enthaltenen Bilder und sonstiges Drittmaterial unterliegen ebenfalls der genannten Creative Commons Lizenz, sofern sich aus der Abbildungslegende nichts anderes ergibt. Sofern das betreffende Material nicht unter der genannten Creative Commons Lizenz steht und die betreffende Handlung nicht nach gesetzlichen Vorschriften erlaubt ist, ist für die oben aufgeführten Weiterverwendungen des Materials die Einwilligung des jeweiligen Rechteinhabers einzuholen.

Weitere Details zur Lizenz entnehmen Sie bitte der Lizenzinformation auf http://creativecommons.org/licenses/by/4.0/deed.de.

